# Fit-failure rate associated with simulated reuse and extended use of N95 respirators assessed by a quantitative fit test

**DOI:** 10.1017/ice.2021.5

**Published:** 2021-01-25

**Authors:** Jiwon Jung, Jiyun Kim, Hyejin Yang, Young-Ju Lim, Sun-Hee Kwak, Min Jee Hong, Eun Ok Kim, Sung-Han Kim

**Affiliations:** 1 Department of Infectious Diseases, Asan Medical Center, University of Ulsan College of Medicine, Seoul, South Korea; 2 Office for Infection Control, Asan Medical Center, Seoul, South Korea

**Keywords:** N95 respirator, fit test, reuse, extended use

## Abstract

**Objective:**

We quantitatively assessed the fit failure rate of N95 respirators according to the number of donning/doffing and hours worn.

**Design:**

Experimental study.

**Setting:**

A tertiary-care referral center in South Korea.

**Participants:**

In total, 10 infection control practitioners participated in the fit test.

**Methods:**

The first experiment comprised 4 consecutive 1-hour donnings and fit tests between each donning. The second experiment comprised 2 consecutive 3-hour donnings and fit tests between each donning. The final experiment comprised fit tests after an 1-hour donning or a 2-hour donning.

**Results:**

For 1-hour donnings, 60%, 70%, and 90% of the participants had fit failures after 2, 3, and 4 consecutive donnings, respectively. For 3-hour donnings, 50% had fit failure after the first donning and 70% had failures after 2 consecutive donnings. All participants passed the fit test after refitting whenever fit failure occurred. The final experiment showed that 50% had fit failure after a single use of 1 hour, and 30% had fit failure after a single use of 2 hours.

**Conclusions:**

High fit-failure rates were recorded after repeated donning and extended use of N95 respirators. Caution is needed for reuse (≥1 time) and extended use (≥1 hour) of N95 respirators in high-risk settings such as those involving aerosol-generating procedures. Although adequate refitting may recover the fit factor, the use of clean gloves and strict hand hygiene afterward should be ensured when touching the outer surfaces of N95 respirators for refitting.

Personal protective equipment (PPE), such as the N95 respirator, is essential for the protection of healthcare workers from severe acute respiratory coronavirus virus 2 (SARS-CoV-2) infection. However, because of the global shortage of N95 respirators arising from the coronavirus disease 2019 (COVID-19) pandemic, extended use (ie, wearing the same N95 respirator for repeated encounters with different patients) or reuse (ie, using the same N95 respirator by one healthcare worker for multiple encounters with different patients but removing after each encounter) of N95 respirators is commonly practiced in many healthcare settings. The Centers for Disease Control and Prevention states that extended use of N95 respirators can be considered in contingency capacity strategies, and limited reuse also can be considered in crisis capacity strategies.^1^ The maximum recommended extended use period of N95 respirators is 8–12 hours. For reuse, if no manufacturer guidance is available, no more than 5 uses per device is recommended.^2^ However, data regarding the safety of reuse and extended use of N95 respirators are limited, and possible reduction in filtration efficiency over time and inadequate seal after multiple donning and doffing are concerning. A recent study performed during the COVID-19 pandemic showed that fit failure was associated with higher number of shifts, donning and doffing, and hours worn^3^; however, the fit test used in this study was qualitative in nature. Therefore, we performed a quantitative fit test to examine the rate of fit failure after extended use and reuse of N95 respirators.

## Methods

This study was performed at Asan Medical Center, a tertiary-care hospital in Seoul, South Korea. Of the 25 infection control practitioners who work at the infection control office of Asan Medical Center, 10 female practitioners volunteered to participate in the experiment. The experiment was performed from June 30 to July 13, 2020. This study was approved by the Asan Medical Center Institutional Review Board (IRB no. 2020-1078).

All quantitative fit tests were conducted using the Portacount Pro+ Respirator Fit Tester 8038 (TSI, Shoreview, MN) in a room (dimensions, 2.8 × 4.6 × 2.7 m^3^) in accordance with the manufacturer’s guideline and the OSHA Respiratory Protection Standards modified ambient aerosol CNC quantitative fit-testing protocol.^4^ The participants engaged in exercises such as bending over, talking, side-to-side movement of the head, and up-and-down movement of the head. The overall fit factor was calculated using an equation provided in the protocol of the Portacount device. The overall fit factor ranged from 0 to 200 and was calculated as the ratio of the concentration of a challenge agent outside the respirator to the concentration of the challenge agent that leaked inside the respirator (C_out_/C_in_). A fit factor of >100 was considered as “passing.” The participants wore 3M 1870+ (3M, Maplewood, MN) because they had previously undergone a quantitative fit test and passed with the 3M 1870+ mask. The particle concentration ranged from 70 to 200. The room temperature was between 24.3°C and 26.5°C and the relative humidity was between 52% and 64%.

**Fig. 1.**
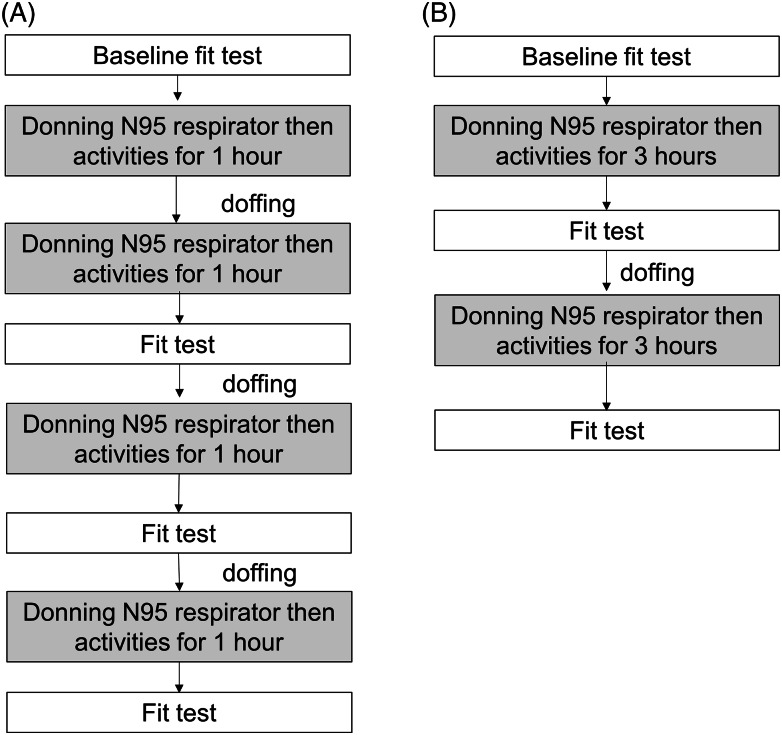
Schematic flow chart of the fit test.

The schematic flow of the experiments is shown in Figure 1. To assess the fit-failure threshold number of reuse, we performed the 2 experiments of 1-hour multiple donning with the same respirator (Fig. 1A) and 3 hours donning with the same respirator (Fig. 1B). If fit failures occurred, refitting by investigator assistance and refit tests were permitted in both experiments. As we observed an unexpectedly high rate of fit failure after 2 consecutive 1-hour donning sessions and a single 3-hour donning session, we additionally performed a third experiment composed of a single 1-hour donning and fit test, and a single 2-hour donning and fit test. While N95 respirators were worn, the participants were not involved in actual clinical care. During the 1-, 2-, and 3-hour trials, they performed routine activities as infection control practitioners including office work with sitting at a desk, phone calls, talking with one another, and walking in the wards. We plugged the hole with tape just after fit test and reused the respirator. We assumed that a punctured mask would not fail the subsequent fit tests.

Graphs with median and interquartile range (IQR) of overall fit factors according to the number of reuses were generated in Prism version 5.01 software (Graphpad Software, La Jolla, CA). Kaplan-Meier curve was generated for the percent of fit failure according to the number of reuses. Normally distributed continuous variables were analyzed using the Student *t* test, and non-normally continuous variables were analyzed using the Mann-Whitney *U* test. All statistical analyses were performed using SPSS Statistics version 21 software for Windows (IBM, Armonk, NY). *P* values < .05 were considered statistically significant.

## Results

**Fig. 2.**
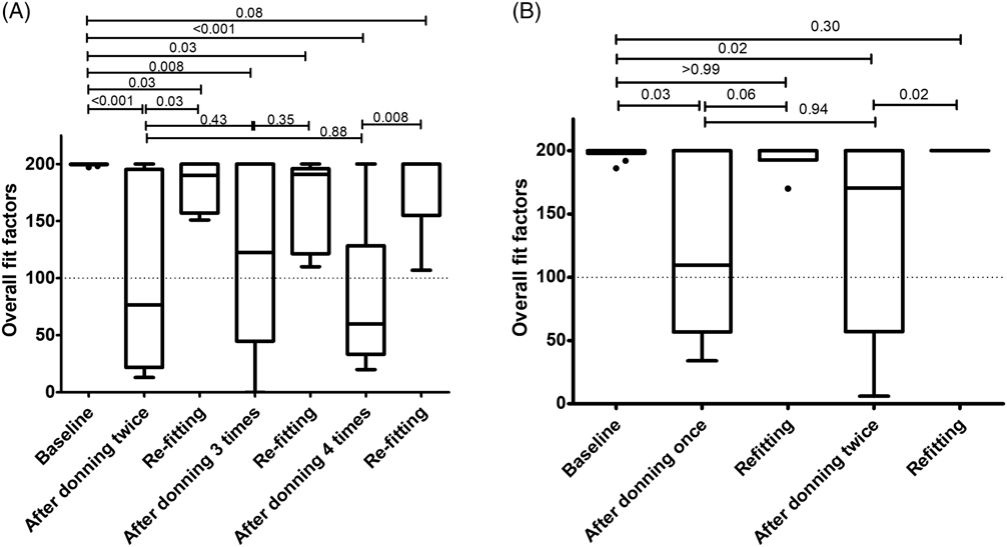
Overall fit factors of the participants at baseline and after repetitive donning/doffing. (A) Results of 1-hour-donning. (B) Results of 3 hours donning. Boxes represent the median and interquartile range [IQR]. Whiskers indicate the upper and lower adjacent values (within 1.5 × IQR), and isolated dots are outlier data points.

**Fig. 3.**
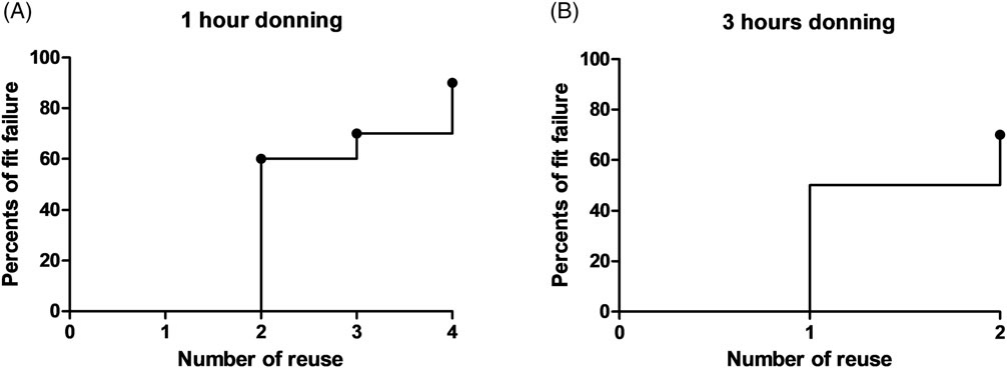
Kaplan-Meier curve for percents of fit failure after repetitive donning and doffing. (A) Fit failure after 1 hour-donning. (B) Fit failure after 3 hours donning. Dots indicate the points of censoring.

In the first experiment of repeated 1-hour donning (Fig. 2A and Table 1), all participants passed at baseline. After 2 consecutive 1-hour donnings, 6 participants had fit failures (median overall fit factor, 77; interquartile range [IQR], 22–195; *P* < .001 vs baseline), and all passed after refitting (median overall fit factor, 190; IQR, 157–200; *P* = .03 vs 2 consecutive 1-hour donnings). After 3 consecutive 1-hour donnings, 5 participants had fit failures (median overall fit factor, 122; IQR, 45–200; *P* = .008 vs baseline), and all passed after re-fitting (median overall fit factor, 191; IQR, 122–196; *P* = .35 vs 3 consecutive 1-hour donnings). After 4 consecutive 1-hour donnings, 7 participants had fit failures (median overall fit factor, 60; IQR, 33–129; *P* < .001 vs baseline), and all passed after refitting (median overall fit factor, 200; IQR, 155–200; *P* = .008 vs 4 consecutive 1-hour donnings). Excluding the result of refitting and censoring when fit failures occurred, 60%, 70%, and 90% of the participants had fit failures after 2, 3, and 4 consecutive 1-hour donnings, respectively (Fig. 3A).

**Table 1.** Results of Repeated 1-Hour Donning Experiment^a^

Participant No.	Baseline	After Donning Twice	Refitting After Donning Twice	After Donning 3 Times	Refitting After Donning 4 Times	After Donning 4 Times	Refitting After Donning 4 Times
1	197	195	…	200	…	**61** ^b^	107
2	200	**87**	159	200	…	184	…
3	200	196	…	200	…	**87**	200
4	200	149	…	169	…	110	…
5	198	**13**	200	176	…	200	…
6	200	**66**	151	**0**	133	**20**	200
7	200	**23**	200	**76**	200	**59**	200
8	200	**26**	200	**29**	191	**37**	197
9	200	**18**	180	**75**	110	**22**	155
10	200	200	…	**50**	192	**41**	200
Median overall fit factor (IQR)	200 (199.5–200)	77 (22–195)	190 (157–200)	122 (45–200)	191 (122–196)	60 (33–129)	200 (155–200)

Note. IQR, interquartile range.

a Overall fit factor of individual participants and the median overall fit factors are shown.

b Bold value indicates fit failure.

In the second experiment of repeated 3-hour donnings (Fig. 2B and Table 2), all participants passed at baseline. After the first 3-hour donning, 5 participants had fit failures (median overall fit factor, 110; IQR, 57–200; *P* = .03 vs baseline), and all passed after refitting (median overall fit factor, 200; IQR, 193–200; *P* = .06 vs 3-hour donning). After 2 consecutive 3-hour donnings, 4 participants had fit failures (median overall fit factor, 171; IQR, 57–200; *P* = .02 vs baseline), and all passed after refitting (median overall fit factor, 200; IQR, 200–200; *P* = .02 vs 2 consecutive 3-hour donnings). Excluding the result of refitting and censoring when fit failures occurred, 50%, and 70% of the participants had fit failures after 1 and 2 consecutive 3-hour donnings, respectively (Fig. 3B).

**Table 2.** Results of Repeated 3-Hour Donning Experiment^a^

Participant No.	Baseline	After Donning Once	Refitting After Donning Once	After Donning Twice	Refitting After Donning Twice
1	200	200		200	
2	200	**100** ^b^	200	145	
3	200	**84**	200	200	
4	200	119	170	**67**	200
5	192	200		196	
6	200	**34**	200	**6**	200
7	200	200		200	
8	200	**63**	200	199	
9	186	**38**	200	**97**	200
10	200	200		**27**	200
Median overall fit factor (IQR)	200 (198–200)	110 (57–200)	200 (193–200)	171 (57–200)	200 (200–200)

Note. IQR, interquartile range.

a Overall fit factor of individual participants and the median overall fit factors are shown.

b Bold value indicates fit failure.

Considering the unexpectedly high rate of fit failure noted in these experiments, we additionally performed fit tests after a single session of 1-hour donning and a single session for 2-hour donning. After 1-hour donning, 5 participants had fit failures (median overall fit factor, 126; IQR, 48–200) (Table 3). After 2-hour donning, 3 had fit failures (median overall fit factor, 160; IQR, 78–185).

**Table 3.** Results of Fit Tests After a Single Use of 1 Hour or 2 Hours

Participant No.	After 1 Hour Donning	After 2 Hours Donning
1	200	155
2	**18** ^a^	170
3	**52**	180
4	**87**	200
5	200	200
6	**63**	**79**
7	200	**76**
8	166	164
9	**34**	**6.5**
10	200	156
Median overall fit factor (IQR)	127 (48–200)	160 (78–185)

Note. IQR, interquartile range.

a Bold value indicates fit failure.

## Discussion

In this study, we performed quantitative fit tests to show that extended use (>1 hour) and reuse of N95 respirators were associated with higher rates of fit failure. Refitting invariably resulted in an appropriate fitting. These data suggest that the extended use or reuse of N95 respirators due to the shortage of N95 respirators and other practical issues should be done with caution, especially in high-risk settings such as those involving aerosol-generating procedures. Therefore, more short-term use or other strategies such as powered air-purifying respirators (PAPR) should be considered in these circumstances.

When caring for patients with COVID-19, healthcare workers usually don PPE during their 3- to 4-hour shifts. Therefore, the extended use of N95 respirators is unavoidable. In addition, because of the ongoing shortage of N95 respirators during the COVID-19 pandemic, the reuse of respirators is common practice in many healthcare settings. Previous studies showed that donning up to 5 times was associated with a relatively low failure rate (<10% and 30%)^5,6^; however, in those studies, the participants wore the N95 respirators for only about 5 minutes, which is far shorter than the usual usage in real-world practice. Therefore, we performed experiments with repetitive 1-hour and 3-hour donning sessions and observed high rates of fit failures.

Presumably, there is a significant risk of airborne transmission of SARS-CoV-2 in aerosol-generating procedures, and the body of evidence on airborne transmission of SARS-CoV-2 is continuing to grow.^7,8^ Our study supports the use of PAPRs during aerosol-generating procedures involving patients with COVID-19 because face-seal leakage could occur during movements of healthcare workers and aerosols can enter inside the N95 respirators through such leakages during extended wearing, although use of PAPRs would be limited at the scale required for the COVID-19 pandemic. Failed fit tests may not directly indicate higher risks of SARS-CoV-2 infection, and the concentration of the challenge agent used in the fit test may not mimic the clinical conditions of caring COVID-19 patients. However, empirical experience warrants that the adequate fit factors should be maintained during the wearing of N95 respirators in healthcare settings until more data are available. Further study is needed to evaluate the association between fit failures of N95 respirators due to extended use or reuse and the risk of SARS-CoV-2 infection.

Although an appropriate fit was achieved in our participants through refitting by healthcare workers, the risk of contact transmission of SARS-CoV-2 from touching a contaminated surface of respirators and subsequently touching facial mucous membranes is greatly concerning. The CDC recommends that healthcare workers use a clean pair of gloves when reusing or adjusting a previously worn N95 respirator and that they discard the gloves and perform hand hygiene after donning or adjusting N95 respirators.^1,2^ Although our data show that refitting restores adequate fit factors, the risk of hand contamination during refitting cannot be overemphasized.

Our study has several limitations. We did not perform these experiments in the setting of patient care for COVID-19 because holes were made in the N95 respirators to insert probes for measurements. Therefore, infection control practitioners performed this study. The activity of healthcare workers caring for patients with COVID-19 may be greater than that of infection practitioners and thus associated with higher rates of fit failure. Also, the participants in this study were highly specialized infection control practitioners who were well educated in mask fitting, and the fitting factors of N95 respirators may be somewhat poorer in nonspecialized healthcare workers. Therefore, the actual fit failure rate may be higher in real-world practice. Notably, although we plugged the hole with tape, the hole might have influenced the subsequent fit-testing results. Lastly, our findings should be interpreted with caution because the rather small number of participants in our study (n = 10) solely consisted of Asian female subjects and because only 1 brand of N95 respirator was tested. Despite these limitations, our study systemically evaluated the quantified fit factors according to the reuse or extended use of N95 respirators, which closely reflects the real-world practice during the COVID-19 pandemic.

In conclusion, the donning and extended use (>1 hour use) of N95 respirators led to higher fit failure rates. The reuse and extended use of N95 respirators should be done with caution in high-risk exposure setting such as those involving aerosol-generating procedures. Although adequate refitting may recover the fit factor, the use of clean gloves and strict hand hygiene afterward should be ensured when touching the outer surfaces of N95 respirators for refitting.
